# *AT2G21280* Only Has a Minor Role in Chloroplast Division

**DOI:** 10.3389/fpls.2017.02095

**Published:** 2017-12-07

**Authors:** Yiqiong Li, Lulu Wang, Guangshuai Wang, Yue Feng, Xiaomin Liu

**Affiliations:** ^1^College of Biological Sciences and Biotechnology, Beijing Forestry University, Beijing, China; ^2^Beijing Key Lab of Bioprocess, College of Life Science and Technology, Beijing University of Chemical Technology, Beijing, China

**Keywords:** chloroplast division, AT2G21280, GC1, AtSulA, phylogenetic analysis

## Abstract

Chloroplast division is an important cellular process, which involves complicated coordination of multiple proteins. In mutant plants with chloroplast division defects, chloroplasts are usually found to be with enlarged size and reduced numbers. Previous studies have shown that *AT2G21280*, which was named as *GC1* (*GIANT CHLOROPLAST 1*) or *AtSulA*, was an important chloroplast division gene, because either reduced expression or overexpression of the gene could result in an apparent chloroplast division phenotype (Maple et al., 2004; Raynaud et al., 2004). To further study the function of *AT2G21280*, we obtained mutants of this gene by CRISPR/Cas9-mediated gene editing and T-DNA insertion. Most of the chloroplasts in the mutants were similar to that of the wild type in size. Larger chloroplasts were rarely found in the mutants. Moreover, we obtained transgenic plants overexpressing *AT2G21280*, analyzed the chloroplast division phenotype, and found there were no significant differences between the wild type and various overexpressing plants. Phylogenetic analysis clearly indicated that AT2G21280 was not in the family of bacterial cell division protein SulA. Instead, BLAST analysis suggested that AT2G21280 is an NAD dependent epimerase/dehydratase family enzyme. Since the main results of the previous studies that *AT2G21280* is an important chloroplast division gene cannot be confirmed by our intensive study and large chloroplasts are rarely found in the mutants, we think the previous names of *AT2G21280* are inappropriate. Localization study results showed that AT2G21280 is a peripheral protein of the inner envelope of chloroplasts in the stroma side. AT2G21280 is well conserved in plants and cyanobacteria, suggesting its function is important, which can be revealed in the future study.

## Introduction

Chloroplasts originated from free-living cyanobacteria as endosymbionts in plant cells (Gould et al., 2008; Keeling, 2013). Like bacteria, chloroplasts are proliferated through binary division, which maintains the stability of the chloroplast number in the cell and is important for the photosynthesis of plants (Dutta et al., 2015). Chloroplast division is carried out by division machinery. Ultrastructural observation showed that there are two plastid dividing (PD) ring structures formed at the division site of chloroplasts: one on the cytosolic surface of the outer envelope membrane (OEM), and the other on the stromal surface of the inner envelope membrane (IEM) (Kuroiwa et al., 1998). The constriction process was suggested to be driven by four different ring-like protein complexes, two in the stroma, the filamenting temperature-sensitive Z (FtsZ) and the inner PD rings, and two in the cytoplasm, the accumulation and replication of chloroplasts 5 (ARC5)/dynamin-related protein 5B (DRP5B) and the outer PD rings (Miyagishima, 2011). With the motive force provided by FtsZ ring and ARC5/DRP5B ring, a chloroplast is divided into two daughter chloroplasts (Yoshida et al., 2006, 2016; Erickson et al., 2010). Chloroplast division occurs in the middle of the organelle. As the first assembled component of division machinery, the localization of FtsZ ring determines the position of the entire division complex (Nakanishi et al., 2009). In *Arabidopsis*, the midplastid localization of FtsZ ring is controlled by the chloroplast Min system, including ARC3, MinD, MinE, and MCD1. The division process of chloroplasts involves a series of proteins, which are assembled into a division complex. Among these proteins, some are derived from cyanobacteria, such as FtsZ1, FtsZ2, ARC6, MinD, and MinE, (Osteryoung, 1995; Colletti et al., 2000; Itoh et al., 2001; Maple et al., 2002; Vitha et al., 2003), while others are of eukaryotic origin, such as ARC5, PDV1 (PLASTID DIVISION 1), and PDV2 (Gao et al., 2003; Miyagishima et al., 2006; Nakanishi et al., 2009). The mutation of chloroplast division genes could result in various chloroplast division phenotypes, such as enlarged dumbbell-shaped chloroplasts, which are due to the mutations in *ARC5*, *PDV1*, or *PDV2* (Pyke and Leech, 1994; Robertson et al., 1996; Gao et al., 2003; Miyagishima et al., 2006), and a few large chloroplasts in the cell, which are due to the mutations in *FtsZ1*, *FtsZ2*, or *ARC6* (Osteryoung et al., 1998; Vitha et al., 2003; Schmitz et al., 2009).

*SulA* is a member of the SOS regulon in *Escherichia coli*. It can partially interfere with cell division followed by a blocking of DNA replication after DNA damage (Huisman and D’Ari, 1981; Huisman et al., 1984). SulA inhibits bacterial cell division by directly interacting with FtsZ and interrupting its normal division activity (Lutkenhaus, 1983; Jones and Holland, 1985; Bi and Lutkenhaus, 1990; Chen et al., 2012; Nazir and Harinarayanan, 2016). This inhibition is reversible because SulA is very unstable and once DNA is repaired, SulA disappears and cell division activity is restored soon after (Mizusawa and Gottesman, 1983; Maguin et al., 1986). In a *lon* mutant, in which the degradation of SulA slows down, the inhibition of cell division by SulA is prolonged (Mizusawa and Gottesman, 1983). As a result, cell morphology is altered with prolonged filamentation (Gottesman et al., 1981).

Previous results suggested that *AT2G21280*, which was named as *GC1* (*GIANT CHLOROPLAST 1*) or *AtSulA*, is an important chloroplast division gene. The protein sequence of AT2G21280 was shown to have a 50% identity with slr1223 protein of *Synechocystis* (SSulA) and ∼65% similarity to All2390 protein of *Anabaena* sp. PCC7120, which were annotated as cell division-inhibitor SulA proteins (Maple et al., 2004; Raynaud et al., 2004). Raynaud et al. (2004) showed that the disruption of *SSulA* in *Synechocystis* caused cell division defect, which could lead to cell death. Then they further showed that in *Arabidopsis*, overexpression of *AtSulA* with a 35S promotor driving full length cDNA with a GFP fusion inhibited chloroplast division in different types of cells, including mesophyll cells, bundle sheath cells and root cells, but the effect of inhibition varied in different lines and even in the same plants. Moreover, it was shown that overexpression of *AtSulA* could restore chloroplast division defect caused by overexpression of *AtFtsZ1-1* or *AtFtsZ2-1* (Raynaud et al., 2004). Maple et al. (2004) found that a severe reduction, but not overexpression, of *GC1* transcripts by cosuppression could cause a strong chloroplast division defect with only a few giant chloroplasts in the cells, whereas the other transgenic plants with normal or elevated level of *GC1* transcripts displayed normal chloroplast division in mesophyll and hypocotyl cells. Therefore, these two studies suggested *AT2G21280* is an important chloroplast division gene but with some contradictions.

To clarify these contradictions and further study the function of *AT2G2180*, we analyzed the chloroplast division phenotype of the mutant and overexpression plants of this gene. In mutant plants, large chloroplasts were observed in rare cases, and the sizes of most of the chloroplasts were found to be similar to that of the wild type. There is no apparent difference of the chloroplast division phenotype between various overexpression plants and the wild-type plants. Furthermore, phylogenetic analysis and sequence analysis indicated that AT2G2180 and SulA are proteins from totally different families. Therefore, our results show that *AT2G2180* is not important for the division of chloroplasts.

## Results

### Knockout of *AT2G21280* Have a Very Little Effect on Chloroplast Division

**FIGURE 1:**
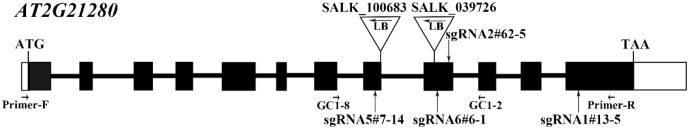
Gene structure of *AT2G21280* and a diagram of T-DNA and sgRNA mutants. White boxes represent the 5′- and 3′-untranslated regions, black boxes represent exons, and black lines represent introns. Triangles and arrowheads in them mark the locations of T-DNA insertion of the SALK lines and indicate the directions of the T-DNA, respectively. Perpendicular arrows mark the targeting sites of sgRNAs. Positions of primers used for T-DNA insertion mutants identification in Supplementary Figure 1 and the RT-PCR analysis in Supplementary Figure 2 are marked with arrows.

**FIGURE 2:**
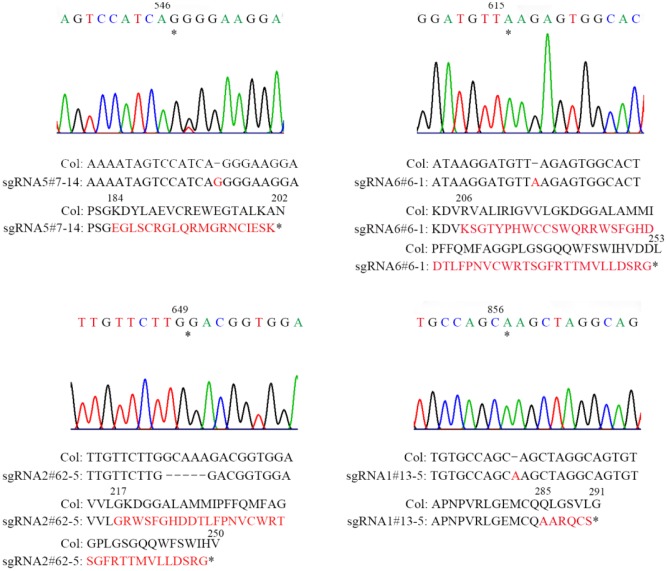
Sequencing analysis of four sgRNA mutants of *AT2G21280*. Asterisks indicate the sites of insertions or deletion in sgRNA mutants. Sequences of DNA and amino acids are compared between the wild type and mutants to show the mutations. Sequences changed are shown in red. Asterisks at the end of amino acid sequences represent stop codon. Numbers above DNA and protein sequences indicated the positions in the cDNA and proteins, respectively.

*AT2G21280* in *Arabidopsis* has 12 exons and 11 introns (**Figure 1**). In order to investigate the function of this gene, we took the advantage of CRISPR/Cas9-mediated gene-editing technique. Four constructs were designed to target four different sites in the gene, respectively and transformed into the wild-type plants. As expected, in the T1 and T2 generation, a part of the transgenic plants were edited at the targeting sites, and sgRNA mutants, such as sgRNA5#7-14, sgRNA6#6-1, sgRNA2#62-5, sgRNA1#13-5 (**Figure 1**), which have mutations in the 8th, 9th, and 12th exons, were obtained (**Figure 2**).

Homozygous mutants were verified by DNA sequencing for further analysis (**Figure 2**). In sgRNA5#7-14, a single base pair was inserted, causing a frame shift from the 184th amino acid and premature termination of the protein soon after. Similarly, in sgRNA6#6-1, a single base pair insertion caused a frame shift from the 206th amino acid and a premature stop codon 46 amino acids downstream. In sgRNA2#62-5, five base pairs were missing, which resulted in a frame shift and premature stop of the protein. In sgRNA1#13-5, a single base pair insertion caused a frame shift and premature stop codon 6 amino acids after. BLAST analysis indicated that the protein sequence of AT2G21280 is well-conserved in plants with 347 amino acids, except the N-terminal region, which is a chloroplast transit peptide. So, the premature stop of the protein in these mutants should have a severe effect on the function of the gene. Even for sgRNA1#13-5, the mutation site is in the last exon and only 63 amino acids upstream of the stop codon, the function of the gene is also very likely to be affected (**Figure 2**).

**FIGURE 3:**
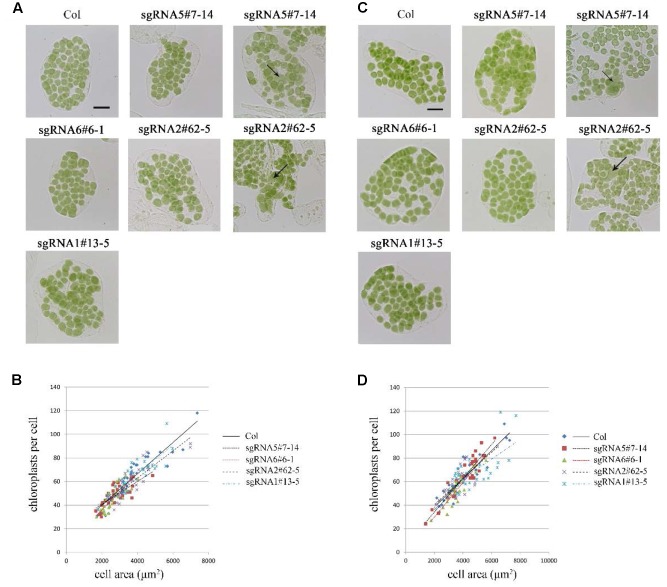
Phenotype analysis of sgRNA mutants. **(A)** Chloroplast division phenotypes of 3-week-old plants. Arrow indicates the enlarged chloroplast. Bar = 20 μm. **(B)** Relationships between chloroplast number and mesophyll cell area in 3-week-old plants. The *R*^2^ values of the best-fit lines are 0.8511, 0.5785, 0.6690, 0.7743, and 0.7173 in order. **(C)** Chloroplast division phenotypes of 5-week-old plants. Bar = 20 μm. **(D)** Relationships between chloroplast number and mesophyll cell area in 5-week-old plants. The *R*^2^ values of the best-fit lines are 0.8274, 0.8586, 0.8003, 0.6238, and 0.4595 in order (*n* = 30).

At first, we studied the chloroplast division phenotypes of the 3-week-old plants of these sgRNA mutants (**Figures 3A,B**). The results indicated that the chloroplast sizes of these mutants are similar to that of the wild type. Only in rare cases, slightly enlarged chloroplasts were found in sgRNA5#7-14 (4 out of more than 800 cells) and sgRNA2#62-5 (1 out of more than 800 cells) (**Figure 3A**). We also analyzed the chloroplast division phenotype of 5-week-old plants, which have larger cell and chloroplast sizes and may give a stronger chloroplast division phenotype. In these mutant plants, chloroplast sizes are also similar to that of the wild type (**Figures 3C,D**). Furthermore, statistical analysis of the numbers of chloroplasts per cell and cell area indicated that there was no obvious difference between the mutant and the wild type, both in 3- and 5-week-old plants (**Figures 3B,D**). This result is completely different from the previous reports that GC1 cosuppression lines contained giant chloroplasts (Maple et al., 2004).

**FIGURE 4:**
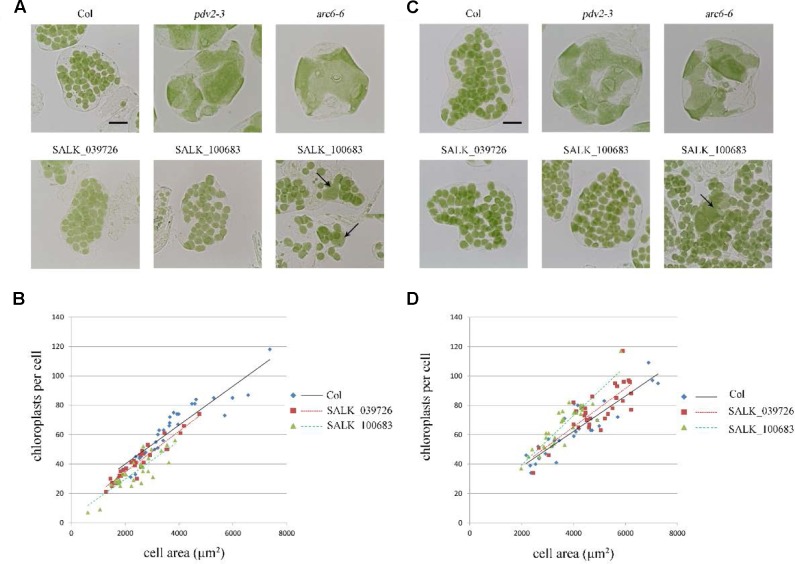
Phenotype analysis of T-DNA mutants. **(A)** Chloroplast division phenotypes of 3-week-old plants of wild type (Col), *pdv2-3*, *arc6-6*, SALK_039726 and SALK_100683. Arrows indicate enlarged chloroplasts in the T-DNA mutants. Bar = 20 μm. **(B)** Relationships between chloroplast number and mesophyll cell area of 3-week-old plants. The *R*^2^ values of the best-fit lines are 0.8511, 0.9017, and 0.7351 in order. **(C)** Chloroplast division phenotypes of 5-week-old plants of wild type (Col), *pdv2-3*, *arc6-6*, SALK_039726 and SALK_100683. Arrow indicates the enlarged chloroplast. Bar = 20 μm. **(D)** Relationships between chloroplast number and mesophyll cell area of 5-week-old plants. The *R*^2^ values of the best-fit lines are 0.8274, 0.6584, and 0.798 in order (*n* = 30).

To further explore this discrepancy, transfer DNA (T-DNA) insertional mutants (SALK_100683 and SALK_039726) were obtained for analysis. The homozygous SALK_100683 and SALK_039726, which contain T-DNA insertions in the 8th exon and the 9th exon, respectively, were verified by PCR (**Figure 1** and Supplementary Figure 1). Mutant plants of SALK_100683 and SALK_039726 at the stages of 3 and 5 weeks were analyzed for the chloroplast division phenotypes (**Figure 4**). We found that the sizes of most of the chloroplasts in the mutants are similar to that of the wild type, and only in rare cases (5 out of more than 800 cells), larger chloroplasts could be found (**Figures 4A,C**). Statistical analysis further indicated that the number of chloroplasts per cell of the mutants and the wild type are similar (**Figures 4B,D**). Thus, the results of T-DNA insertion mutants are similar to that of sgRNA mutants. At the same time, chloroplast division mutants *pdv2-3* and *arc6-6* were used as controls for comparison (Vitha et al., 2003; Miyagishima et al., 2006; Chang et al., 2017; Wang et al., 2017). These mutants contains only a few giant chloroplasts in the cell (**Figures 4A,C**).

The transcriptional level of *AT2G21280* in different sgRNA and T-DNA mutant lines was analyzed by semi-quantitative reverse transcription (RT) PCR (Supplementary Figure 2). The results showed that the levels of *AT2G21280* were reduced in all of these mutant lines. Especially, PCR product was undetectable in T-DNA insertion mutants.

**FIGURE 5:**
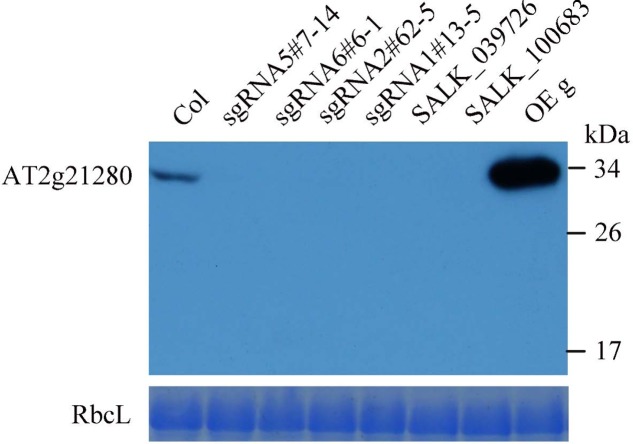
Western blot analysis of AT2G21280. The specificity of the antibodies and the protein level of AT2G21280 in the Col wild type, sgRNA mutants, T-DNA mutants and overexpression plant were analyzed. Total proteins were extracted from leaves of 4-week-old plants, and loaded in each lane. RbcL was used as a loading control. The molecular weight of protein markers are labeled on the right.

To further analyze the protein levels in these mutant lines, we generated the antibodies of AT2G21280. As shown in **Figure 5**, a band of approximately 33 kD was detected in the wild type, which is close to the expected size of AT2G21280. While in all of these mutants, this band was missing. Moreover, no band smaller than this size was detected in these mutants. These results indicated that the protein of AT2G21280 was either not translated or degraded. Therefore, these sgRNA and T-DNA mutants are true knockout mutants.

Taken together, these results showed that *AT2G21280* only has a minor role in chloroplast division.

### Overexpression of *AT2G21280* Has No Effect on Chloroplast Division

**FIGURE 6:**
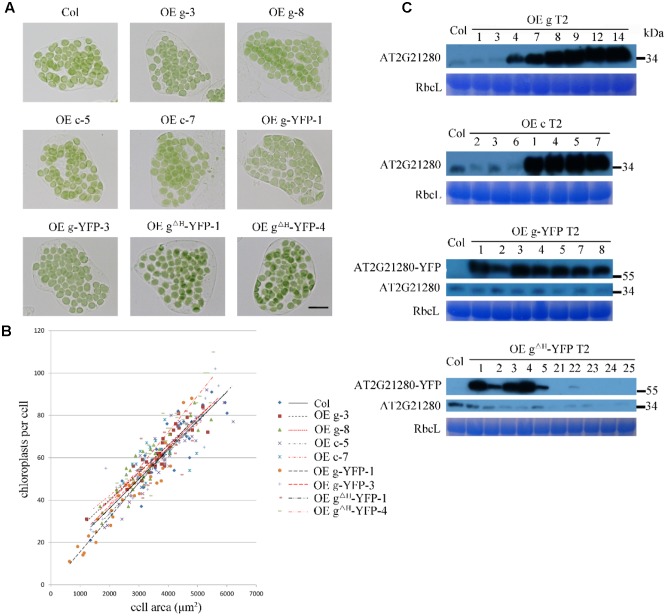
Phenotype and protein level analysis of AT2G21280 overexpressing plants. **(A)** Chloroplast division phenotypes of different overexpression lines. “OE g” represents the overexpression of genomic DNA, “OE c” represents the overexpression of cDNA, “OE g-YFP” represents the overexpression of AT2G21280 fused with YFP, “OE g^ΔH^ -YFP” represents the overexpression of AT2G21280 lacking the last 20 amino acids with a C-terminal YFP fusion. Leaf tissues were sampled from 4-week-old plants. The scale bar is 20 μm. **(B)** Relationships between chloroplast number and mesophyll cell area of the wild type and different AT2G21280 overexpressing plants. The *R*^2^ values of the best-fit lines are 0.7814, 0.8314, 0.6333, 0.8096, 0.8238, 0.9101, 0.8457, 0.6875, and 0.8840 in order (*n* = 30). **(C)** Immunoblot analysis of the protein level of AT2G21280 in different plants. Total proteins were extracted from leaves of 4-week-old plants, and loaded in each lane. RbcL served as a loading control. The molecular weight of protein markers are labeled on the right.

To test whether overexpression of *AT2G21280* can affect chloroplast division as reported before (Raynaud et al., 2004), we transformed *Arabidopsis* wild-type plants with constructs containing CaMV35S-driven full-length cDNA (c), or full-length genome DNA (g) individually. Moreover, we also obtained transgenic plants expressing 35S-g-YFP and 35S-g^ΔH^-YFP, respectively. The latter had a truncation of the last C-terminal 20 amino acids (for details see below) of the protein. The protein level of AT2G21280 in the transgenic plants with various constructs as mentioned above was analyzed by immuno-blot. Most of the transgenic plants have a protein level much higher than that of the wild type (**Figure 6C**). We chose the plants with a very high level of AT2G21280 for phenotypic analysis. The chloroplasts in these plants are very similar to those in the wild-type plants (**Figure 6A**). Then we analyzed chloroplast division phenotypes of these plants by statistical analysis and still found no obvious differences between these overexpression lines and the wild type (**Figure 6B**).

In addition, in some of the lines, the endogenous protein level of AT2G21280 was undetectable, possibly due to cosuppression. The chloroplast phenotype of these lines was also analyzed (Supplementary Figure 3). Phenotypic and statistical analysis results indicated that there was no significant difference between them and the wild type.

Thus, the previous report that overexpression of AtSulA could cause a chloroplast division defect (Raynaud et al., 2004) cannot be verified by us. Based on these results and the results shown above, we think the previous names of *AT2G21280*, *GC1* and *AtSulA*, are not appropriate.

### AT2G21280 Is Localized to the Envelope of Chloroplasts

**FIGURE 7:**
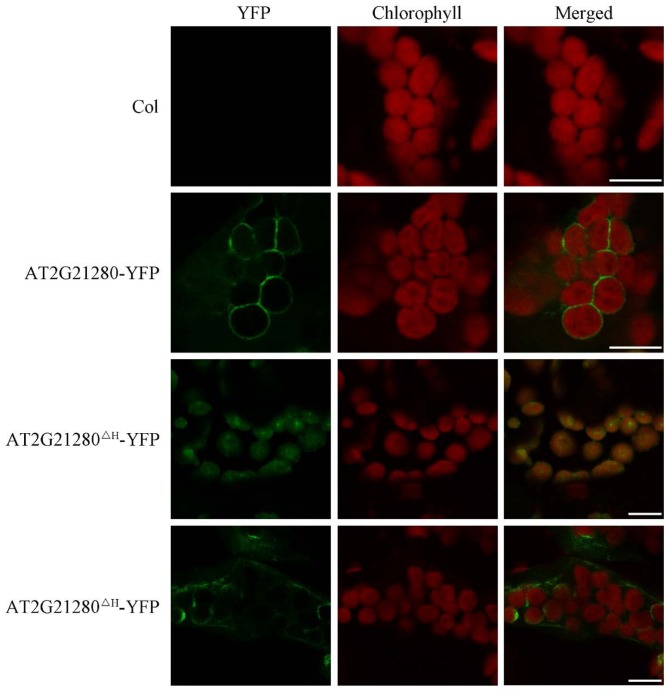
Subcellular localization study of AT2G21280-YFP and AT2G21280^ΔH^-YFP. The full length of AT2G21280 or a truncation of the last 20 AA at C-terminal end (AT2G21280^ΔH^) with a YFP fusion were used to study the subcellular localization of the protein in *Arabidopsis*. Col wild type was used as a control. Green fluorescence signals indicate AT2G21280-YFP or AT2G21280^ΔH^-YFP, and red signals indicate the autofluorescence of chlorophyll. Bar = 10 μm.

Previous study suggested that the nine amino acids at the C-terminal end is an amphipathic helix which may anchor AT2G21280 to the chloroplast inner envelope in tobacco leaf cells with a transiently expression of *35S-AT2G21280-YFP* fusion protein (Maple et al., 2004). We studied the subcellular localization of AT2G21280 with GFP fused to the full-length protein 35S-g-YFP (or AT2G21280-YFP), and a protein with a 20 amino acids truncation at the C-terminal end, 35S-g^ΔH^-YFP (or AT2G21280^ΔH^-YFP) in *Arabidopsis*. The results showed that, consistent with previous results, AT2G21280-YFP was indeed localized to the envelope of chloroplasts (**Figure 7**). However, we found that AT2G21280^ΔH^-YFP had two types of distribution, one is dot-like aggregation in the stroma as before (Maple et al., 2004), the other is a region near the chloroplast envelope, which is wider than and different from that of AT2G21280-YFP (**Figure 7**), suggesting when missing the last C-terminal 20 amino acids, AT2G21280 cannot bind well to the envelop membrane. The latter localization result is different from the previous study (Maple et al., 2004).

### Phylogenetic Analysis of AT2G21280 and SulA

Previous results suggest that AT2G21280 is similar to slr1223 protein of *Synechocystis* (SSulA) and All2390 protein of *Anabaena* sp. PCC7120, which were annotated as SulA homologs in *Cyanobacteria* (Maple et al., 2004; Raynaud et al., 2004). These proteins are well-conserved in *Cyanobacteria* and plants. However, a BLAST search with the real SulA protein of *E. coli* found no homologs in *Cyanobacteria* and plants. Moreover, BLAST searches with AT2G21280, slr1223, All2390, or their homologs all suggest these proteins are NAD dependent epimerase/dehydratase family enzymes, which is totally different from SulA.

**FIGURE 8:**
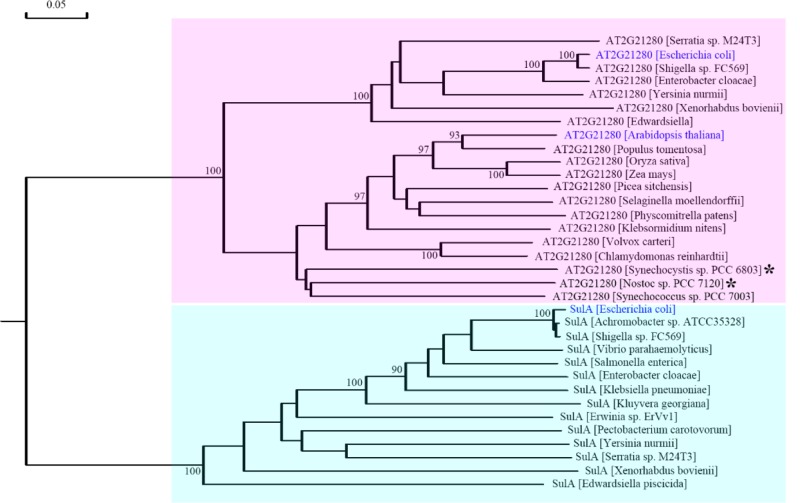
Phylogenetic analysis of AT2G21280 and SulA. A phylogenetic tree of the protein sequences of AT2G21280 and SulA and their homologs in different species. Asterisks indicated the species whose sequences were used to search homologous proteins in *Arabidopsis* and found AT2G21280 in previous studies. Anabeana was later renamed as Nostoc. AT2G21280 in *Arabidopsis*, its homologs in *E. coli*, and SulA in *E. coli* are shown with blue color. AT2G21280 and SulA families are highlighted with pink and cyan colors, respectively. Bootstrap values ≥ 90% are shown at the corresponding nodes based on 1000 bootstrapping replicates.

To resolve this problem, we retrieved homologous sequences of SulA in bacteria and AT2G21280 in bacteria and plants based on BLAST search results and carried out a phylogenetic analysis. The result clearly indicated that SulA and AT2G21280 are proteins of distinct families (**Figure 8**). AT2G21280 belongs to a family widely distributed in bacteria and plants, while SulA belongs to a family in *E. coli* in its close relatives. Therefore, it is a mistake to name AT2G21280 as AtSulA.

## Discussion

An optimized number and size of chloroplasts is important for the normal physiological function of chloroplasts *in vivo* (Dutta et al., 2015). Therefore, plant cells must maintain an appropriate division of chloroplasts. Chloroplast division is completed by a protein complex composed of many components. If one protein of the complex is severely affected by mutation or other types of interference, it can cause abnormal chloroplast division, and result in a reduction of chloroplast number and an enlargement of chloroplast size (Osteryoung et al., 1998; Miyagishima et al., 2006) (**Figure 4A**). In this study, we analyzed the role of *AT2G21280*, a gene previously reported as a chloroplast division gene, in chloroplast division. By observing and statistically analyzing the chloroplast division phenotype of *at2g21280* mutants and *AT2G21280* overexpressing plants, we found that *AT2G21280* is not important for chloroplast division. Moreover, bioinformatic analysis suggested that there is no relationship between AT2G21280 and bacterial cell division-related protein SulA.

Previous studies reported that AT2G21280 is a chloroplast division protein. Maple et al. (2004) found that AT2G21280 was localized to the stromal side of chloroplast inner envelope by the C-terminal amphipathic helix. They also obtained AT2G21280 transgenic *Arabidopsis* plants with a CaMV35S-driven full-length cDNA in the sense orientation. They reported that a greatly reduction of *AT2G21280* transcript level could result in a reduction of the number of chloroplasts and the occurrence of giant chloroplasts in the mesophyll cells of the cosuppression lines, while a great increase of *AT2G21280* transcript level had no effect on chloroplast division. Furthermore, cosuppression of *AT2G21280* produced homogenously giant chloroplasts in mesophyll cells but chloroplasts with heterogeneous sizes in hypocotyl cells, especially in the cells closed to the hypocotyl base (Maple et al., 2004). Raynaud et al. (2004) reported loss of function mutation of *SSulA* in *Synechocystis* by gene disruption affected cell division. Furtherly, they constructed *AT2G21280-GFP* with full length cDNA of *AT2G21280* under control of the 35S promotor in sense orientation. They screened transgenic plants through observing GFP fluorescence in root cells. Their analysis suggested that overexpression of *AT2G21280* could cause an obvious chloroplast division defect in some cells and the phenotype was heterogeneous even in the same plant. They suggested the inhibition of plastid division was due to the high levels of AT2G21280-GFP (Raynaud et al., 2004). In contrast, Maple et al. (2004) found increased level of *AT2G21280* had no effect on chloroplast division in *Arabidopsis*. That is, these studies have some contradictions.

In our study, we analyzed the role of AT2G21280 in chloroplast division with several knockout mutants, including RNA-guided CRISPR/Cas9 mutants, T-DNA insertion mutants and possible AT2G21280 cosuppression lines in *Arabidopsis*. In addition, we obtained various overexpression lines, including overexpression the full length genomic DNA or cDNA of *AT2G21280*, and *YFP* fusion genes. Our Western Blot results also showed the protein levels in the mutant or transgenic plants have undetectable or very high levels of AT2G21280. This kind of experiments are more convincing but were not carried out in the previous studies (Maple et al., 2004; Raynaud et al., 2004). Nevertheless, our genetic and phenotypic analysis studies with multiple lines of evidences showed that enlarged chloroplasts in the mutant can only be found in rare cases, and overexpression of AT2G21280 doesn’t affect chloroplast division. These results are generally quite different from the previous reports.

AT2G21280 in *Arabidopsis* was found by searching the homologous proteins of the bacterial cell division-inhibitor protein SulA. It shared ∼65% similarity to with the *Anabaena* sp. PCC 7120 All2390 (Maple et al., 2004). But our experimental results do not support the conclusion that AT2G21280 is a chloroplast division protein. Therefore, is the *Anabaena* sp. PCC 7120 All2390 a real SulA protein? What is the relationship between AT2G21280 in *Arabidopsis* and SulA in *E. coli*. A phylogenetic analysis of AT2G21280-related proteins and SulA-related proteins was carried out to resolve these questions. Our analysis clearly shows that AT2G21280 and SulA are from different families (**Figure 8**). Therefore, they should have different functions in chloroplasts or bacterial cells.

Subcellular localization in both previous study and our analysis showed that AT2G21280 was localized to the inner envelope of chloroplasts. Then, what is the real function of AT2G21280? BLAST search result suggested that AT2G21280 is mostly composed of a conserved domain of NAD (P)-dependent epimerase or an atypical short-chain dehydrogenase, which use nucleotide-sugar substrates for a variety of chemical reactions. Sugar epimerase is widely found in animals, plants and microorganisms. It is initially isolated from *E. coli*, catalyzing an epimerization reaction through the transient reduction of NAD^+^ (Arabshahi et al., 1988; Somers et al., 1998). Once an epimerase gene of bacteria is mutated, it can cause reduced infection, decreased pathogenicity, and high sensitivity to antibiotics (Coleman and Leive, 1979; Vaara, 1993; Yamashita et al., 1999). Based on these facts, we speculate that AT2G21280 may modify and regulate the carbohydrates attached to the surface of the membrane of chloroplast inner envelope in the stromal side.

## Materials and Methods

### Plant Materials and Growth Conditions

All *Arabidopsis* plants used in this study are in Col ecotype background. T-DNA insertion mutants of *AT2G21280*, SALK_039726 and SALK_100683, were obtained from ABRC (Arabidopsis Biological Resource Center, United States). Seeds were sterilized and sowed on 1/2MS (Murashige and Skoog) solid medium containing 0.8% agar and 1% sucrose. After being placed in a refrigerator at 4°C for 2 days, plates were moved to a growth chamber at 22°C with 16-h-light/8-h-dark cycles. Ten days later, seedlings were transferred into soil and grown in the same growth chamber.

### Chloroplast Phenotype and Fluorescence Microscopy Analysis

Leaf fixation and chloroplast phenotype analysis were performed as described previously (Gao et al., 2013; Chang et al., 2017). Briefly, a piece of 4-week-old leaf was immersed into a tube containing 1 mL 3.5% glutaraldehyde for 1 h in the dark. Then the fixative solution was replaced with 0.1 M Na_2_EDTA (pH9.0) and the tube was incubated in water bath at 55°C for 2 h. Chloroplast phenotype was observed with an Olympus CX21 microscope (Olympus, Tokyo, Japan) equipped with a USB 2.0 digital camera (Changheng, Beijing, China). Statistical analysis of chloroplast phenotypes was done as before (Gao et al., 2013). Fluorescence images of YFP and chlorophyll were obtained with a TCS SP8 confocal laser scanning microscope (Leica Microsystems, Germany).

### Plasmid Construction

A series of constructs were made based on the CRISPR/CAS9 plasmid, pHEE401E (Wang et al., 2015), in order to edit *AT2G21280*. Targeting sites were chosen with the tools at the website CRISPRscan^1^ and CAS-OFFinder^2^. Primers used for targeting four different sites of *AT2G21280* (sgRNA5, sgRNA6, sgRNA2, and sgRNA1) were shown in Supplementary Table 1.

For constructs overexpressing *AT2G21280*, OE g (genomic DNA) and OE c (cDNA), the full length genomic DNA and cDNA were amplified by PCR with primers 2g21280-5 and 2g21280-6 (Supplementary Table 1), digested with *Nco*I and *Mlu*I, and cloned into 3302Y2 vector, respectively.

To construct 35S-g-YFP and 35S-g^ΔH^-YFP, full length genomic DNA (g) of *AT2G21280* and *AT2G21280* lacking of the last 20 amino acids at the C terminus were amplified using primers GC1-5 and GC1-6 and GC1-5 and GC1-7 (Supplementary Table 1). The PCR products digested with *Mlu*I were cloned into pCAMBIA 3302Y3 vector.

### Generation of Transgenic Plants

Overexpressing constructs were introduced into *Agrobacterium tumefaciens* and then transformed into *Arabidopsis* by floral dipping method (Feldmann and Marks, 1987; Clough and Bent, 1998; Bent, 2006). Transgenic plants of T1 generation were screened with Basta. T2 plants were used for the analysis.

Transgenic plants of *AT2G21280* sgRNA were obtained as described above. The T1 transgenic plants were screened on 1/2 MS medium containing 20 μg/mL hygromycin and 10 μg/mL carbenicillin. The plates were placed in the dark for 3∼4 days and then moved to the light. One week later, seedlings selected by the antibiotic were transferred into soil and grown in a growth chamber.

### Identification of sgRNA Mutants and T-DNA Insertion Mutants

For *AT2G21280* sgRNA mutants, sgRNA5#7-14, sgRNA6#6-1, and sgRNA2#62-5 were identified by amplifying genomic DNA sequences using primers GC1-8 and GC1-2 and analyzing the sequencing results of the PCR products (Supplementary Table 1). For sgRNA1#13-5, primers GC1-9 and GC1-3 were used instead (Supplementary Table 1).

The sequences flanking the insertion sites of T-DNA mutants, SALK_039726 and SALK_100683, were amplified by PCR with primers GC1-8 and LBC1. The accurate insertion sites were deduced by DNA sequencing. The genomic DNA sequence spanning the insertion sites in the two T-DNA mutants was amplified by primers GC1-8 and GC1-2 (Supplementary Table 1 and **Figure 1**).

### RNA Extraction and RT-PCR Analysis

RNA was isolated from the leaves of 4-week-old plants grown in soil under white light, using an RNApure Total RNA Isolation Kit (Aidlab, Beijing, China). The RNA samples (3 μg each) were used as templates for first-strand cDNA synthesis (Thermo Fisher Scientific, United States). Semi-quantitative RT-PCR analysis was performed according to Pan et al. (2013). *AT2G21280* were amplified with specific primers GC1-5 and GC1-7. The *PP2AA3* gene was taken as a control and amplified with primers PP2AA3-1 and PP2AA3-2.

### Immunoblot Analysis

To generate AT2G21280 antibodies, a fragment of AT2G21280, which is from the 46th amino acid to the end, was expressed in *E. coli* and purified as antigen. Antibodies were produced in rabbit and purified. For immunoblot analysis, proteins from 5 mg of leaves were separated by SDS–PAGE and transferred to PVDF membrane (Bio-rad). After being blocked with 5% milk for 2 h, the PVDF membrane was incubated with anti-AT2G21280 polyclonal antibodies at a dilution of 1:1000 in 3% milk for 1 h, then washed with 3% milk for four times, and incubated with HRP-labeled goat anti-mouse IgG secondary antibody at a dilution of 1:10,000. Finally, an eECL Western Blot kit (Beijing ComWin Biotech Company, China) were used for the film development.

### Phylogenetic Analysis

Homologous sequences of AT2G21280 and SulA in various species were searched with NCBI BLAST^3^ and downloaded (Supplementary Table 2). Phylogenetic analysis of AT2G21280, SulA and their relatives was carried out by DNAman software (Version 7, Lynnon Biosoft Inc., United States).

## Author Contributions

XL conceived the project and designed the experiments. YL, LW, and GW performed most of the experiments. YF participated in the preparation of AT2G21280 antibodies. XL and YL wrote the manuscript. All authors read and approved the final manuscript.

## Conflict of Interest Statement

The authors declare that the research was conducted in the absence of any commercial or financial relationships that could be construed as a potential conflict of interest.

## Footnotes

1 http://www.crisprscan.org/?page=sequence

2 http://www.rgenome.net/cas-offinder/

3 https://blast.ncbi.nlm.nih.gov/Blast.cgi
