# Supplementary material for: AT2G21280 Only Has a Minor Role in Chloroplast Division
Source: Front Plant Sci. 2017 Dec 7;8:2095. doi: 10.3389/fpls.2017.02095 (PMC5725473; doi:10.3389/fpls.2017.02095)
Supplement: Supplementary file 1 [file Data_Sheet_1.docx]

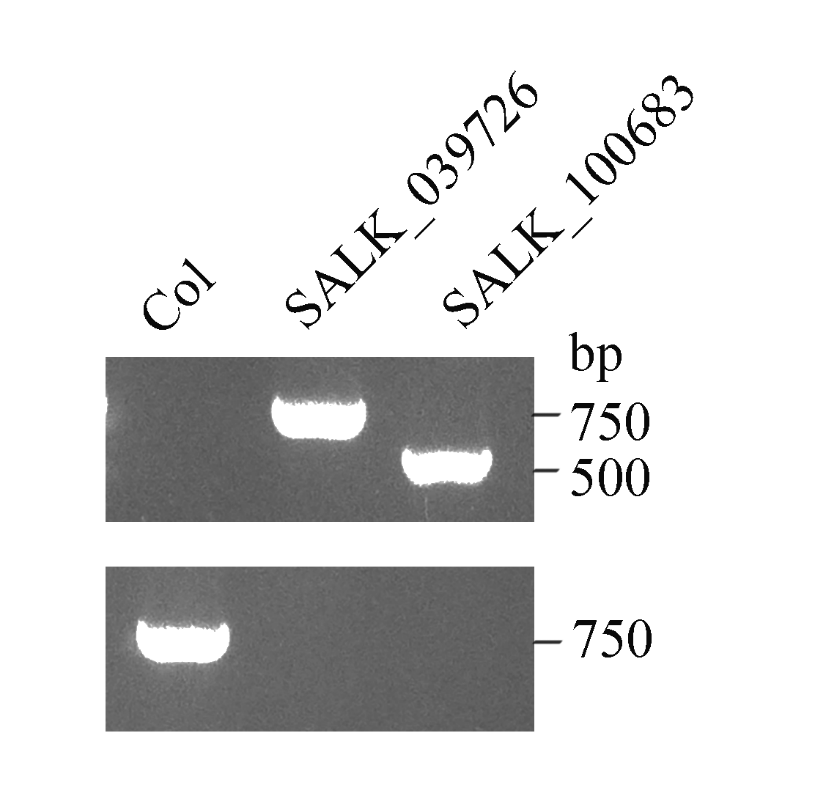


**Supplemental Figure 1** PCR identification of T-DNA insertion mutants.

The wild-type DNA and the flanking sequences of T-DNA mutants were amplified by PCR and run on agrose gels. Sizes of DNA ladders are marked on the right. Positions of T-DNA insertion and the primers used for the analysis are shown in **Figure 1**.


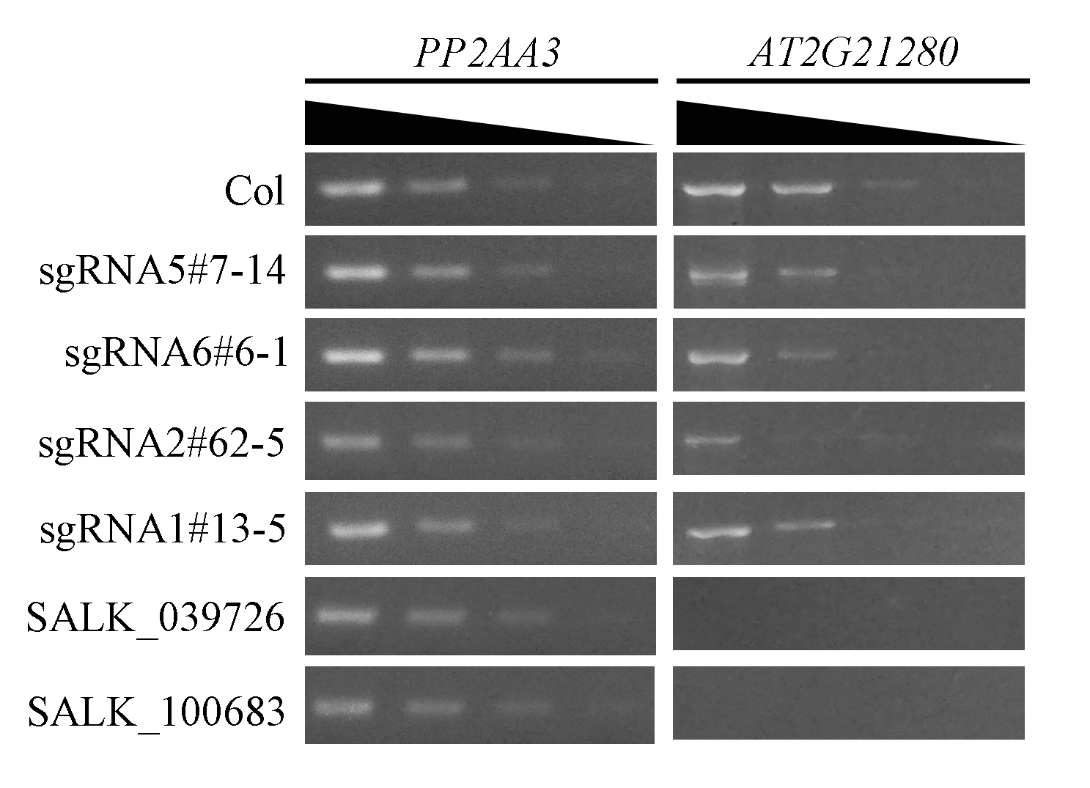


**Supplemental Figure 2** RT-PCR analysis of *AT2G21280*. Col wild type, four sgRNA mutants and two T-DNA mutants were analyzed. Positions of the primers used for RT-PCR analysis are indicated in **Figure 1**. Black triangles indicate that the quantity of cDNA was serially diluted three times with a dilution factor of 4 (from left to right). *PP2AA3* was used as a control.


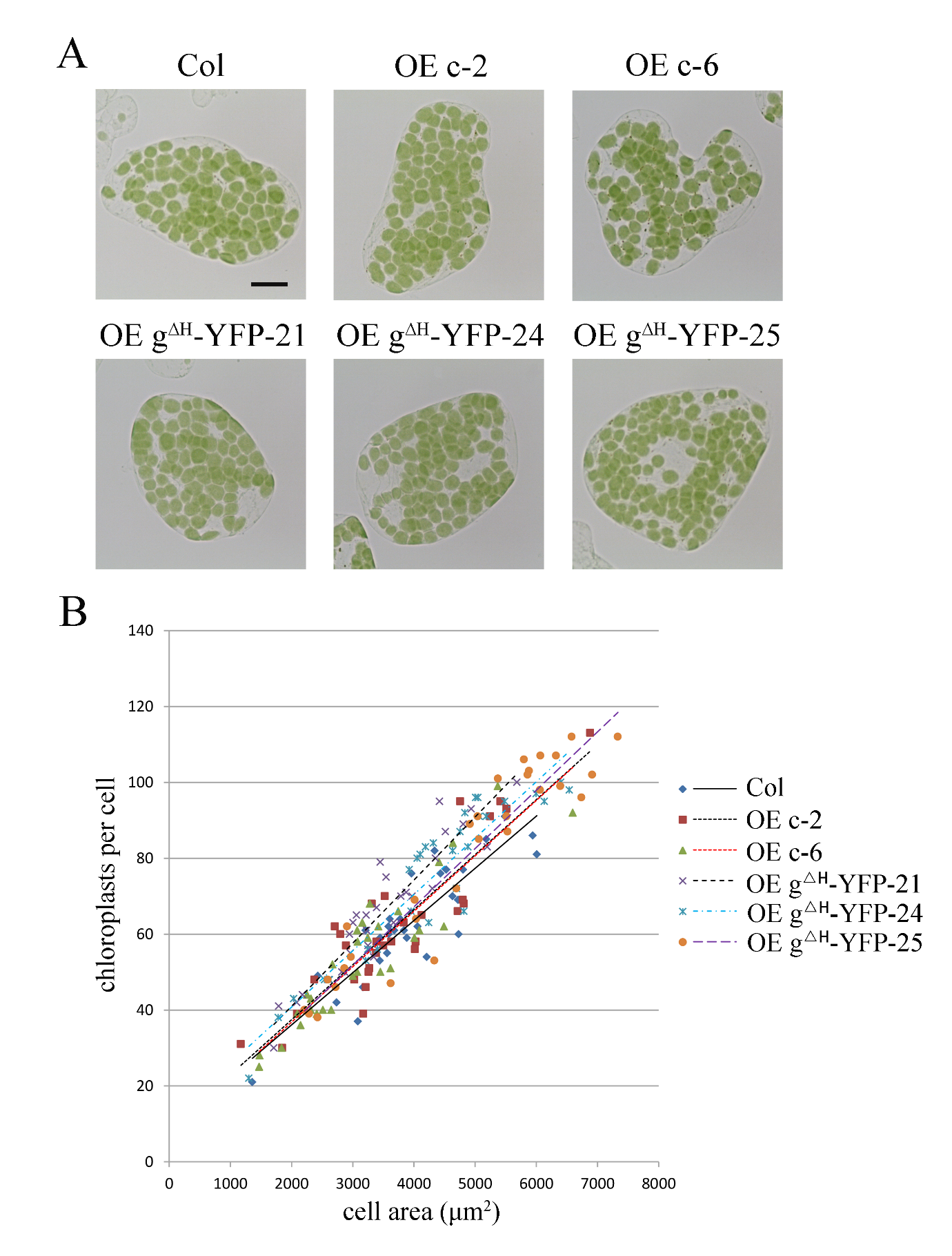


**Supplemental Figure 3** Phenotype analysis of putative AT2G21280 cosuppression lines as shown in Figure 6. **(A)** Chloroplast division phenotypes of 3-week-old plants of Col, OE c-2, OE c-6, OE g^△H^-YFP-21, OE g^△H^-YFP-24 and OE g^△H^-YFP-25. Bar = 20 μm. **(B)** Relationships between chloroplast number and mesophyll cell area of 3-week-old plants. The R^2^ values of the best-fit lines are 0.7814, 0.8095, 0.8543, 0.8814, 0.8747, 0.9056 in order (n = 30).

**Supplemental Table 1** Primer sequences used in this study.

| primer | sequence |
| --- | --- |
| GC1-2 | 5'- TGG AAA AAG GGT ATC ATC ATG GC -3' |
| GC1-3 | 5'- CTG AGA TAA CAC CGA AAA CAC TTT TTA CC -3' |
| GC1-5 | 5'- CAC CAC TCA CTG TTA ACG ATC -3' |
| GC1-6 | 5'- CCG ACG CGT TTG CAT AAT GGC TCT GAG TG -3' |
| GC1-7 | 5'- CCG ACG CGT TTT GGC TCT TAC AGG TAA TAC C -3' |
| GC1-8 | 5'- CCT CCA AAG TTA GTG ATT ACT GTC -3' |
| GC1-9 | 5'- GTG TTG TTC TTG GCA AAG AC -3' |
| 2G21280-5 | 5'- CCT CCA TGG AGC TTC TCT GCT CAC C -3 |
| 2G21280-6 | 5'- CCT ACG CGT GGG GAT TTT CTG TGA TCA AAA CC -3' |
| PP2AA3-1 | 5'- CCA AGC GGT TGT GGA GAA C -3' |
| PP2AA3-2 | 5'- GAA CCA AAC ACA ATT CGT TGC TG -3' |
| LBC1 | 5'- CGA TTT CGG AAC CAC CAT CAA ACA GG -3' |
| Oligo-01-F5 | 5'- ATTG GAA AAT AGT CCA TCA GGG A -3' |
| Oligo-R5 | 5'- AAAC TCC CTG ATG GAC TAT TTT C -3' |
| Oligo-01-F6 | 5'- ATTG GCA AAT AAG GAT GTT AGA G -3' |
| Oligo-R6 | 5'- AAAC CTC TAA CAT CCT TAT TTG C -3' |
| Oligo-01-F2 | 5'- ATTG GTG TTG TTC TTG GCA AAG A -3' |
| Oligo-R2 | 5'- AAAC TCT TTG CCA AGA ACA ACA C -3' |
| Oligo-01-F1 | 5'- ATTG GGG AAA TGT GCC AGC AGC T -3' |
| Oligo-R1 | 5'- AAAC AGC TGC TGG CAC ATT TCC C -3' |

**Supplemental Table 2** The corresponding organisms and NCBI accession numbers of the sequences used in the phylogenetic analysis in Figure 7.

| Organism | NCBI accession number | Family |
| --- | --- | --- |
| *Arabidopsis thaliana* | NP_001324767.1 | AT2G21280 |
| *Populus tomentosa* | APR64415.1 | AT2G21280 |
| *Oryza sativa* | XP_015623635.1 | AT2G21280 |
| *Zea mays* | NP_001336960.1 | AT2G21280 |
| *Picea sitchensis* | ABK23230.1 | AT2G21280 |
| *Selaginella moellendorffii* | XP_002993609.1 | AT2G21280 |
| *Physcomitrella patens* | XP_001766815.1 | AT2G21280 |
| *Klebsormidium nitens* | GAQ93156.1 | AT2G21280 |
| *Volvox carteri* | XP_002953146.1 | AT2G21280 |
| *Nostoc* sp. PCC 7120 | WP_010996546.1 | AT2G21280 |
| *Synechococcus* sp. PCC 7003 | WP_065714150.1 | AT2G21280 |
| *Synechocystis* sp. PCC 6803 | BAA17507.1 | AT2G21280 |
| *Chlamydomonas reinhardtii* | XP_001702195.1 | AT2G21280 |
| *Serratia* sp. M24T3 | WP_009636444.1 | AT2G21280 |
| *Escherichia coli* | WP_077577632.1 | AT2G21280 |
| *Enterobacter cloacae* | WP_049137259.1 | AT2G21280 |
| *Yersinia nurmii* | WP_049597011.1 | AT2G21280 |
| *Edwardsiella* | WP_012849234.1 | AT2G21280 |
| *Xenorhabdus bovienii* | WP_038218788.1 | AT2G21280 |
| *Shigella* sp. FC569 | WP_069372407.1 | AT2G21280 |
| *Escherichia coli* | ABE06509.1 | SulA |
| *Salmonella enterica* | WP_080249355.1 | SulA |
| *Kluyvera georgiana* | WP_064548862.1 | SulA |
| *Achromobacter* sp. ATCC35328 | CUK10410.1 | SulA |
| *Vibrio parahaemolyticus* | KKF70677.1 | SulA |
| *Klebsiella pneumoniae* | WP_085327978.1 | SulA |
| *Erwinia* sp. ErVv1 | WP_067702953.1 | SulA |
| *Pectobacterium carotovorum* | WP_039275674.1 | SulA |
| *Shigella* sp. FC569 | ODQ05543.1 | SulA |
| *Enterobacter cloacae* | CZX04881.1 | SulA |
| *Yersinia nurmii* | WP_049596474.1 | SulA |
| *Xenorhabdus bovienii* | WP_012987351.1 | SulA |
| *Serratia* sp. M24T3 | WP_037378119.1 | SulA |
| *Edwardsiella piscicida* | GAJ66555.1 | SulA |
